# Decrease in Mortality after the Implementation of a Hospital Model to Improve Performance in Sepsis Care: Princess Sepsis Code

**DOI:** 10.3390/jpm14020149

**Published:** 2024-01-29

**Authors:** Rosa Méndez, Angels Figuerola, Fernando Ramasco, Marta Chicot, Natalia F. Pascual, Íñigo García, Andrés von Wernitz, Nelly D. Zurita, Auxiliadora Semiglia, Alberto Pizarro, Carmen Saez, Diego Rodríguez

**Affiliations:** 1 Department of Anaesthesiology and Surgical Intensive Care, Hospital Universitario de La Princesa, Diego de León 62, 28006 Madrid, Spain; fernando.ramasco@salud.madrid.org; 2 Department of Preventive Medicine and Public Health, Hospital Universitario de La Princesa, Diego de León 62, 28006 Madrid, Spain; angels.figuerola@salud.madrid.org; 3 Department of Intensive Care Medicine, Hospital Universitario de La Princesa, Diego de León 62, 28006 Madrid, Spain; marta.chicot@salud.madrid.org; 4 Department of Clinical Analysis, Hospital Universitario de La Princesa, Diego de León 62, 28006 Madrid, Spain; nataliafernanda.pascual@salud.madrid.org; 5 Department of General Surgery, Hospital Universitario de La Princesa, Diego de León 62, 28006 Madrid, Spain; inigo.garcia@salud.madrid.org; 6 Department of Emergency, Hospital Universitario de La Princesa, Diego de León 62, 28006 Madrid, Spain; andres.vonwernitz@salud.madrid.org (A.v.W.); alberto.pizarro@salud.madrid.org (A.P.); 7 Department of Microbiology, Hospital Universitario de La Princesa, Diego de León 62, 28006 Madrid, Spain; nellydaniela.zurita@salud.madrid.org (N.D.Z.); mariaauxiliadora.semiglia@salud.madrid.org (A.S.); 8 Department of Internal Medicine, Hospital Universitario de La Princesa, Diego de León 62, 28006 Madrid, Spain; carmenmaria.saez@salud.madrid.org; 9 Department of Intensive Care Medicine, Hospital Universitario Príncipe de Asturias, Avenida Principal de La Universidad s/n, 28805 Madrid, Spain; diego-anibal.rodriguez@salud.madrid.org

**Keywords:** sepsis code, sepsis, septic shock, mortality, intensive care units, qSOFA

## Abstract

Sepsis is a time-dependent disease whose prognosis is influenced by early diagnosis and therapeutic measures. Mortality from sepsis remains high, and for this reason, the guidelines of the Surviving Sepsis Campaign recommend establishing specific care programs aimed at patients with sepsis. We present the results of the application of a hospital model to improve performance in sepsis care, called *Princess Sepsis Code*, with the aim of reducing mortality. A retrospective study was conducted using clinical, epidemiological, and outcome variables in patients diagnosed with sepsis from 2015 to 2022. A total of 2676 patients were included, 32% of whom required admission to the intensive care unit, with the most frequent focus of the sepsis being abdominal. Mortality in 2015, at the beginning of the sepsis code program, was 24%, with a declining rate noted over the study period, with mortality reaching 17% in 2022. In the multivariate analysis, age > 70 years, respiratory rate > 22 rpm, deterioration in the level of consciousness, serum lactate > 2 mmol/L, creatinine > 1.6 mg/dL, and the focus of the sepsis were identified as variables independently related to mortality. The implementation of the Princess Sepsis Code care model reduces the mortality of patients exhibiting sepsis and septic shock.

## 1. Introduction

Sepsis is defined as a life-threatening organ dysfunction secondary to the host’s dysregulated response to infection [1]. Sepsis is a significant threat due to it’s the high incidence in the population and its high impact on hospital mortality, along with its costs in to the environment [2].

Sepsis exhibits a time-dependent pathology with a high mortality, reaching around 10% in the case of sepsis, and rising above 40% in patients with septic shock [1]

It is estimated that there are 189 cases of sepsis treated in hospitals per 100,000 person-years, with a mortality rate of 26.7% [3]. The estimated incidence of sepsis requiring admission to the medical and surgical intensive care units (ICU) was 30%, of which 41.9% died before hospital discharge.

The pathophysiology of sepsis is currently unknown. Advances and discoveries are continually occurring, leading to new theories regarding the way in sepsis affects the organism, depending on circumstances and phenotypes. For this reason, it is difficult to develop specific treatments, as the literature results are often disparate, and there are no currently no strategies or drugs showing positive results in reducing mortality [4].

Therefore, while continuing to seek improvements in precision medicine, one of the strategies for improving sepsis outcomes is the implementation of structured performance improvement programs in sepsis care, based on organization, education, and access to diagnostic and therapeutic resources [5].

Every 4 years since 2004, updates to the guidelines of the Surviving Sepsis Campaign (SSC) have been published, and these present the best available evidence for the management of sepsis. These are universal guidelines, developed using GRADE methodology, and they include recommendations and suggestions for the management of these patients. Since the quality of the evidence and the results regarding sepsis and septic shock in the literature are not promising, most of the recommendations have a low level of evidence and rely on the expression of expert opinions and best practices. Nonetheless, these guidelines are excellent tools as the basis for clinical practice.

The first recommendation in the latest SSC guidelines [6] is as follows:

“For hospitals and health systems, we recommend using a performance improvement program for sepsis, including sepsis screening for patients with acute and high-risk illnesses and standard operating procedures for treatment. Strong Recommendation.”

In 2015, a model for improving performance in sepsis, called the Princess Sepsis Code (PSC), was developed at La Princesa University Hospital in Madrid, based on Peter Pronovost’s model of cultural change [7] and the conviction that improvement based on packages of measures had already been incorporated into clinical practice, as large clinical trials in sepsis had shown [8,9,10,11,12].

A multidisciplinary team including doctors from different specialties, nurses, clinical documentation, and informatics was created to develop PSC, with an annual rotating direction between the different services and shared leadership.

PSC was defined as a program to improve performance in sepsis, providing the hospital’s healthcare workers with a set of clinical, analytical, microbiological, and organizational tools which, together with the intense educational activity of all health personnel, aimed to diagnose the patient early and start the best treatment as quickly as possible in order to decrease mortality [13].

**Figure 1:**
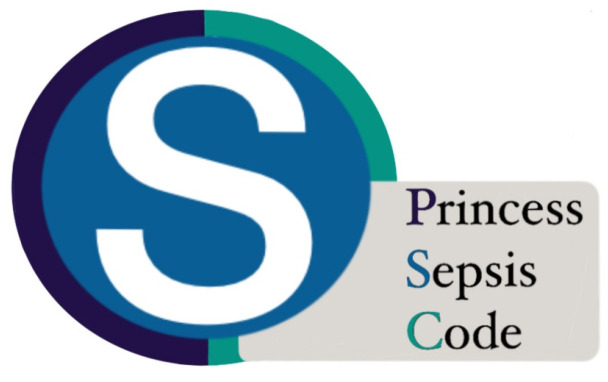
Princess Sepsis Code logo.

A logo was designed for PSC, under which all clinical and educational activities were carried out (Figure 1).

**Figure 2:**
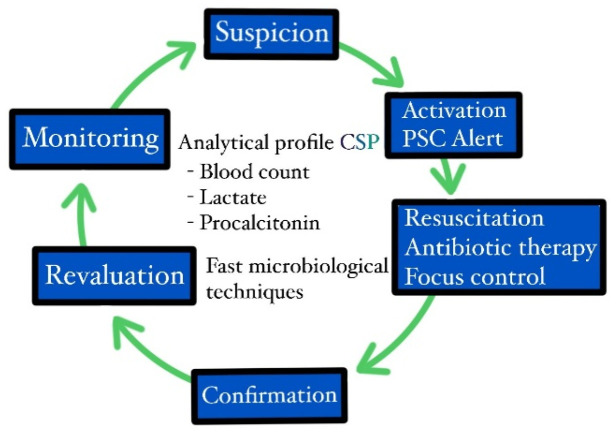
Action algorithm of the Princess Sepsis Code.

From a practical point of view, when sepsis or septic shock are suspected (based on scales such as “quick SOFA” (qSOFA) or the systemic inflammatory response syndrome (SIRS)) or confirmed (based on the sequential organ failure assessment (SOFA) score), the clinician in charge of the patient activated the PSC alert in the computer system, and from that moment on, the patient received priority care, including rapid analytical, microbiological testing (determining the causal microorganism in approximately 1 h, which is routinely determined in 12–24 h, and its antibiogram in 7 h, which is routinely determined in 72–96 h); rapid radiological testing; the initiation of treatment with resuscitation and antibiotic therapy; the control of the infectious focus in an invasive manner, by surgical intervention or interventional radiology; and assessment and admission to the medical and surgical ICU, if the evolution continued to be unfavorable, despite treatment (Figure 2).

All these measures were universally extended in the hospital through a continuous educational program aimed at first-year resident doctors, nursing and emergency care doctors, hospitalization wards, and ICUs, through online courses on an e-learning platform, blended courses that based their practical portion on high-realism simulation and cognitive aids, among other initiatives.

The type of program employed in our hospital was endorsed by different published studies that confirmed its effectiveness, in a global manner and regarding specific aspects, during these years, and it was also endorsed by multidisciplinary recommendations that supported its implementation in a hospital environment [14,15,16,17].

The main objective of this study is to evaluate the impact of the PSC program on mortality during the years between 2015 and 2022.

## 2. Materials and Methods

This is a retrospective observational analytical study of all patients receiving a PSC alert activation due to the diagnosis of sepsis or septic shock from 1 February 2015 to 31 December 2022. During this period, the definition of sepsis changed, but since only patients with at least one organ failure were included in the PSC alert in the previous period, this determination coincided with the updated definition of sepsis.

Among the clinical–epidemiological variables, age, sex, systolic blood pressure (SAP), and heart rate (HR) were collected, with respiratory rate (RR), oxygen saturation (SpO_2_), level of consciousness, and temperature added in 2017, all of which were obtained at the time of PSC alert activation. Among the analytical parameters, lactate, procalcitonin, and creatinine values were analyzed. Regarding the hemodynamic parameters, vasoconstrictor and inotropic drugs administered in the first 6 h were recorded. Blood cultures collected when activating the PSC alert were reviewed, as well as antibiotics received by the patient in the first 6 h after activation. Severity factors included ICU admission and the SOFA scale. Finally, the outcome variables were 30-day mortality and days of ICU stay.

For the descriptive analysis of qualitative variables, frequency and percentage were determined using the χ^2^ test or Fisher’s non-parametric test for comparison. For the quantitative variables, the mean and its standard deviation (SD) were calculated, comparing them with the analysis of variance (ANOVA), the Student’s *t*-test, or the non-parametric Mann–Whitney U test.

To determine the variables independently associated with mortality, a logistic regression (LR) model was constructed among those variables with a statistically significant association in the bivariate analysis, calculating the corresponding adjusted OR of each.

Finally, the associations between all variables and mortality, according to the origin of sepsis, were analyzed using LR, calculating the adjusted OR in each of the groups.

Statistical significance was set at *p* < 0.05. Analyses were performed using SPSS Version 19 Statistical Software.

The study was approved by the clinical studies committee of the University Hospital of La Princesa, with registration number PI-893.

## 3. Results

A total of 2676 patients presented with PSC alert activation throughout the study years: 232 in 2015, 193 in 2016, 287 in 2017, 395 in 2018, 371 in 2019, 322 in 2020, 389 in 2021, and 487 cases in 2022.

In 78% of cases, PSC was activated in the emergency department, 8% in the ICU, and 14% in the hospitalization ward. A total of 32% of the patients analyzed required admission to the ICU, with an average stay in these units of 3 ± 10 days, and an increase was observed over the years of the study.

**Figure 3:**
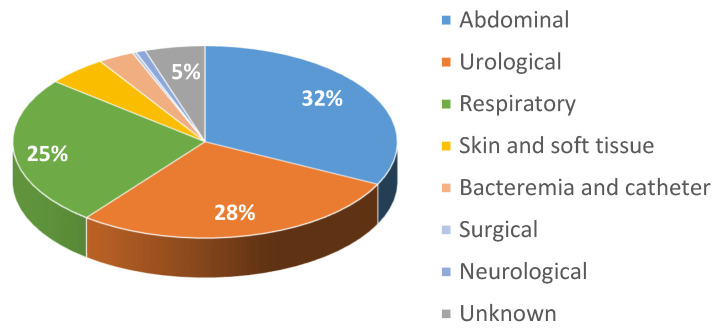
Location of the origin of sepsis infection.

The mean age of the study population was 74 ± 15 years, and 60% of the participants were male. Regarding the septic focus, the most frequent was abdominal (32%), followed by urological (28%), and respiratory (25%), with 5% of unknown origin (Figure 3). Blood cultures were taken in 79% of cases, and 34% were positive for sepsis. In the first 6 h after PSC activation, 27% of patients required vasoactive drugs, and 96% of patients received antibiotic therapy: 84% in the first hour after PSC activation, 13% between 1 and 3 h, and 3% between 3 and 6 h.

Table 1 shows the differences observed in the main variables analyzed during these years.

**Figure 4:**
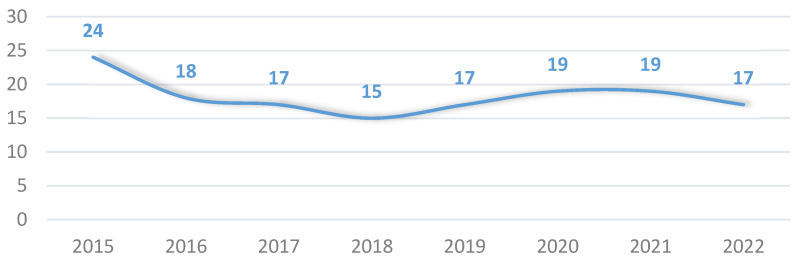
Evolution of percentage of mortality throughout the years of the study.

In the study, the overall mortality was 18%, indicating a 7% decrease in mortality since the start of the project, from 24% in 2015 to 17% in 2022. However, these differences were not statistically significant (Figure 4).

The mortality rate of patients with PSC activation in the ICU was 24%, 17% for those with PSC activation in the emergency department, and 21% for those with PSC activation in the hospitalization ward. These differences were statistically significant, i.e., if the patient was activated in the ICU, the risk of dying was 1.6 times higher than if the patient was activated in the emergency room, and if the patient was activated in the hospitalization ward, the risk of dying was 1.3 times higher than for those activated in the emergency room (*p* < 0.05).

The highest mortality rate was detected among sepsis episodes originating from skin and soft tissue infections (25%), followed by those originating with a respiratory focus (23%), sepsis of unknown origin or without focus (21%), sepsis with an abdominal focus (19%), bacteremia related or non-catheter related sepsis (13%), and sepsis with a urinary focus (11%), with sepsis of neurological origin expressing the lowest mortality rate (10%). These differences were statistically significant (*p* < 0.05).

In the bivariate analysis, the following were associated with mortality: age, sex, focus of origin of sepsis, SAP, RR, SpO_2_, impaired level of consciousness, serum lactate levels, creatinine at the time of activation, the need to administer amines in the first 6 h of PSC activation, activation outside the emergency department, failure to collect blood cultures in the first hours of septic symptoms, and non-administration of antibiotics in the first 6 h after activation.

In the multivariate logistic regression (LR) analysis of mortality, in which those variables with a statistically significant association in the bivariate analysis were included, age > 70 years, RR > 22 rpm, impaired level of consciousness, serum lactate > 2 mmol/L, creatinine > 1.6 mg/dL at the time of activation of the PSC alert, and the focus of origin of sepsis were identified as independent variables associated with mortality (Table 2).

The variables associated with mortality were analyzed according to the most frequent foci of origin of sepsis: abdominal, urinary, and respiratory.

When analyzing abdominal sepsis in the entire series (846 cases), we identified age > 70 years, deterioration in the level of consciousness, serum lactate > 2 mmol/L, creatinine > 1.6 mg/dL at the time of activation of the PSC alert. the administration of amines during the first 6 h and the need for admission to the ICU (Table 3).

In urinary sepsis (752 cases), we identified the deterioration of the level of consciousness and serum lactate > 2 mmol/L at the time of activation of the PSC alert as variables associated with mortality (752 cases), using multivariate LR analysis (Table 4).

In sepsis of respiratory origin (674 cases), the variables independently associated with mortality were: age > 70 years, deterioration in the level of consciousness, and the need to administer amines during the first 6 h after activation of the PSC (Table 5).

Patients were classified into four groups according to the SOFA score, the distribution of which was as follows: 87% of patients had a score equal to or less than 6 (with a mortality of 15%), 9% between 7 and 9 (with a mortality of 34%), 3% between 10 and 12 (with a mortality of 50%), and 1% exceeded 12 points (with a mortality of 67%). A linear association between the SOFA score and the risk of dying was detected, and the risk was 3 times higher if the SOFA score was between 7 and 9, compared to the risk if the SOFA score was ≤ 6 group, 6 times greater if the SOFA score was between 10 and 12, and 11 times higher if the SOFA score exceeded 12 points.

**Figure 5:**
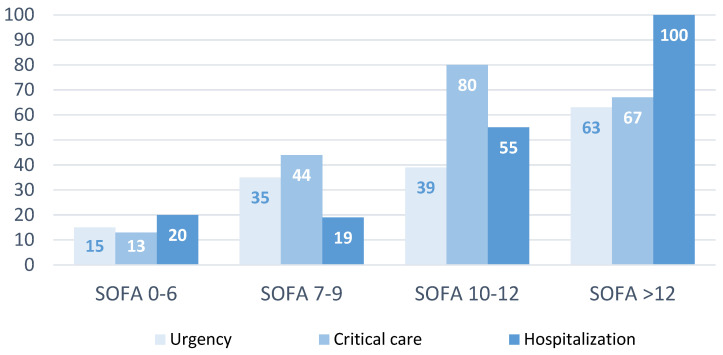
Mortality according to SOFA group and activation department.

Figure 5 shows the percentage of mortality according to the SOFA scale and the department in which the PSC activation was carried out. It should be noted that patients with SOFA scores higher than 12 whose PSC alerts were activated on the hospitalization floor exhibited a 100% mortality rate.

When constructing a variable with the presence or absence of the prognostic factors included in the qSOFA score (SAP < 100 mmHg, RR > 22, and deterioration of the level of consciousness), in order to assess its efficacy in the discrimination of severity, the patients were classified into four groups: 5% did not present any of the prognostic factors, and the mortality rate was 4%; 24% exhibited one of the prognostic factors, with a mortality rate of 9%; 49% showed two of the factors, with a mortality rate of 21%; and 22% expressed the three factors analyzed, with a mortality of 36%. A linear association was detected between having or not having any of these factors and mortality, with the mortality rate being 2 times higher if one of the factors was present, compared if none were noted; 6 times greater if two factors were present; and 13 times higher if the three prognostic factors of qSOFA were exhibited.

## 4. Discussion

The implementation of PSC as an organizational and educational model for improving performance in the care of patients with sepsis and septic shock decreases mortality, with the following variables being independently associated: age, increase in RR, deterioration in the level of consciousness, elevation in lactate and creatinine levels, and the source of sepsis.

Research on how to improve performance in the treatment of sepsis has recently been considered one of the most relevant aspects for investigation. For this reason, studies such as this one are relevant [18].

In 2016, the new definitions of sepsis and septic shock were published in the journal *JAMA* [1], referring to a mortality rate of around 10% for sepsis, which rose above 40% in the case of septic shock. Likewise, the main studies including patients with sepsis [8,9,10] placed the mortality rate for sepsis and septic shock at between 20% and 30%.

In 2015, a population-based study was published in Spain that included more than 240,000 patients diagnosed with sepsis and septic shock during the years 2006 to 2011 [19]. The study found a mortality rate of 43%, with an increase in incidence rate over the years, mainly related to age and associated comorbidities, highlighting the need for a new approach in regards to diagnostic and treatment measures.

In our study, the mortality rate during the first year of implementation of the PSC organizational model was 24%, a figure that decreased to 17% in 2022, reflecting a downward trend.

In 2021, our group published a study including patient mortality data from 2015 to 2018 [15]. It showed a statistically significant linear decrease in mortality from 24% in 2015 to 15% in 2018. In the present study, we observed a slight oscillation in mortality between 17 and 19% over the last four years. There are several possible explanations for this oscillation, which include some interesting aspects to highlight.

The COVID-19 pandemic led to a decrease in face-to-face training for healthcare personnel; nevertheless, good results in terms of mortality were maintained, probably thanks to continuing with the specific online training regarding the PSC on an e-learning platform [20,21].

On the other hand, following the theory of Peter Pronovost [7], the continuous replacement of personnel, along with new advances in diagnosis and treatment, require a maximum level of educational standards that must be maintained over time in such a way that a higher level of both organizational and educational actions would be necessary to improve results [22].

In this regard, Damiani et al. [23] demonstrated that performance improvement programs were associated with increased adherence to SSC-promoted packages of measures and reduced mortality in patients with sepsis and septic shock. Interventions that included both an educational program and process changes were associated with greater benefit in terms of survival. It seems clear that quality improvement initiatives, such as PSC, represent a valuable tool to promote the best care for patients with sepsis. This meta-analysis also highlighted the importance of educating the medical and nursing staff, as it demonstrated that education alone could improve performance, and it was associated with reduced mortality. That is why PSC includes education as one of its fundamental pillars.

Over the last few years, numerous studies have been published regarding the results of sepsis and septic shock when implementing packages of measures and organizational models similar to PSC. Muhtadi et al. [24] presented a series of 4000 patients in which they conducted a comparison before and after instituting a sepsis care improvement program, finding a 5% decrease in sepsis mortality and a 6.5% decrease in septic shock mortality, as well as improved adherence to package measures. In our study, we do not have data on sepsis mortality before the implementation of PSC, but we did discover a decrease in mortality since the beginning of the program, in figures similar to those reported by these authors, along with a good compliance with the items in the SSC measure packages. In addition, our working group published a study in 2022 comparing the mortality of patients admitted to the internal medicine ward in whom the PSC alert was activated or not, detecting a significantly higher mortality at 28 days in patients where the PSC alert was not activated compared to patients with PSC activation (43% vs. 21%) [16].

In the same vein, there are numerous studies with positive morbidity and mortality results, thanks to the implementation of a sepsis care model, in the form of either a rapid response team or multidisciplinary groups organized in a similar way to those employed in the PSC [25,26,27,28,29].

Of particular note is a study published in *Critical Care* by Schinkel et al. [30]. In this study, the authors pointed to two main considerations:

(a) Poor adherence to the measures recommended by the SSC guidelines, which is why it was necessary to implement action programs such as PSC, was noted. They referred to the fact that, despite being measures endorsed by more than 30 scientific societies at an international level, the lack of scientific evidence and the timing to implement each measure continued to be a topic of debate. The question they asked was, “Is one-size-fits-all sufficient in the treatment of sepsis”? And the answer was that most of the measures included in the care packages contributed positively to the management of the disease in the majority of patients. In PSC, the package of measures was based on the recommendations of the SSC guidelines adapted to the hospital’s logistics, insisting not only on the importance of early intervention, but also on constant re-evaluation, given that the course of sepsis is dynamic. In addition, as a possible substitute for the high adherence of all healthcare personnel to PSC measures, patient activation, which remained constant in each year of the study, was observed.

(b) Having a sepsis care program, in itself, already brought about an improvement in results, regardless of the specific bundles of each package of measures. This is because, when implementing the sepsis care program, health personnel were more attentive to these patients and understood this pathology as time-dependent, with a high risk of mortality. In our environment, thanks to the establishment of PSC and the intense training activity, health personnel associated sepsis with a pathology dependent on early diagnosis and treatment. Therefore, blood cultures were extracted in 79% of patients with PSC activation, 96% received antibiotic therapy in the first 6 h (84% were administered in the first hour after activation of the PSC alert), and the use of vasoactive drugs, when necessary, was initiated in the first 6 h after activation. In accordance with the latest recommendations in the hemodynamic management of sepsis, which emphasized the early use of vasoconstrictors (to avoid an excess volume of fluids), administered in the first hours, and to ensure perfusion [31,32]. It is also important to remember the classic study by Kumar et al. [33], which showed that, for every hour of delay in the initiation of antibiotic treatment, mortality increased by 7.6%, distinguishing the delay from the presence of hypotension.

On the other hand, in our study, 32% of patients required admission to the ICU, which is lower than that found in the literature, with percentages of around 75–80% [8,9]. This could be related to the availability in the emergency room and in the hospitalization ward of the necessary tools for the early diagnosis and treatment of sepsis, thanks to the PSC program, as well as to the appropriate training of medical personnel.

No differences were found in the mortality rate of patients who required admission to the ICU during their stay compared to those who did not, which is another factor in favor of the correct management of patients in the emergency room and in the hospitalization ward, probably related to the cross-sectional and formative nature of the PSC model, since what is usually referenced in the literature is a higher mortality rate among patients who required admission to the ICU.

In our study, we found that the most frequent focus of sepsis was abdominal, followed by urological and respiratory; however, this is not common, with the respiratory focus being the most frequent focus noted in clinical series and trials [2,34,35].

The factors that may have influenced the main focus of sepsis at the abdominal level were: the significant involvement of the surgical staff with the PSC model and the fact that the hospital where the study was carried out was eminently surgical, with a high rate of both surgical and endovascular digestive procedures.

It has been reported that in-hospital mortality due to sepsis differs according to the cause of infection, suggesting that the focus is important with respect to prognosis, as evidenced in our results, where the focus is an independent prognostic variable with respect to mortality [35].

It is especially relevant to investigate the differences and factors related to mortality depending on the focus, as it can help us to understand different phenotypes, in view of an increasingly individualized case management, which has significance as a new line of future research for the PSC group to look for the differences in mortality between surgical and non-surgical patients, along with variables related to outcomes in surgical patients [36,37,38].

A noteworthy result of our study, due to its prognostic implications, is the high mortality of the most seriously ill patients, diagnosed by a higher score on the SOFA scale, mainly in those activated in the hospitalization ward. A study by Christa Schorr [39] describing the results of a performance improvement program detected, as in our series, a higher mortality rate in patients with sepsis diagnosed in the ward compared to those diagnosed in the emergency department, which is the reason why they started a sepsis care program. Possible causes included delays in diagnosis and treatment and a lack of staffing. This is one of the areas in which the PSC group is working to improve results.

There are multiple studies on risk factors associated with mortality in patients with sepsis [40,41,42]. In all of them, the following are mainly defined as risk factors for mortality: age, associated comorbidities, previous use of antibiotics, degree of organ dysfunction, increase in the levels of some analytical markers (such as lactate, creatinine, and procalcitonin), and the type of sepsis and focus of origin. In our study population, the risk factors associated with mortality were age over 70 years, respiratory rate greater than 22 rpm, impaired level of consciousness, serum lactate greater than 2 mmol/L, creatinine greater than 1.6 mg/dL, and origin of sepsis. Biomarkers are increasingly important in personalized medicine in regards to the treatment of sepsis; in this sense, lactate and creatinine level are universally employed, although they have their limitations [43].

It is noteworthy that hemodynamic variables were not independently related to mortality, except if the focus was abdominal or respiratory. There is controversy in the literature concerning this topic, probably because there are different hemodynamic phenotypes associated with different prognoses, according to the management performed [44,45].

Similarly, the administration of the antibiotic in the first 6 h after PSC activation was not significantly associated with mortality, despite the high degree of compliance with this measure in our sample of patients. The state of the art is early administration [6], but there is also published literature that questions the excessive precocity of antibiotic administration [46]. The results of antibiotic therapy are influenced by numerous circumstances, such as appropriate treatment or pharmacokinetics, which define precocity as one more piece of a complex puzzle [47].

Diagnostic scales are key to improve sepsis performance in a hospital program, and this was highlighted by the definition “Sepsis 3” when, in parallel to this label, a simple marker such as qSOFA was defined for universal use [1]. There are numerous studies in this field that are in favor of the use of the National Early Warning Score (NEWS) or a scale similar to the qSOFA; however, its use as a diagnostic scale in isolation is not recommended in the latest EFS guidelines due to its low sensitivity [6,48,49]. Choices should therefore be based on preferences and possibilities at the local level. In our PSC model, SIRS and qSOFA were used jointly to carry out the suspected diagnosis, in anticipation of implementing the MEWS scale in the hospital’s computer system in the future. However, our results with the use of qSOFA were positive, probably because the activation of a qSOFA alert was invariably linked to an assessment of the patient by the specialist and to the assessment of other clinical and laboratory data as a whole, such as by employing procalcitonin [50]. In our experience, it could be said that qSOFA became a detection scale for clinical deterioration, which led to the search for more immediate data for the diagnosis of sepsis. Likewise, the prognostic value of the qSOFA scale was very noteworthy in our results, detecting a higher mortality in patients who, at the time of PSC activation, exhibited the three positive factors of the scale. Its ease of implementation makes it a useful tool in an era of limited resources [51]. Very interesting studies have been published regarding the use of automatic alerts for the detection of sepsis, and the use of artificial intelligence, big data, and machine learning in this context, which, although in the preliminary phases for clinical use, are tools that should be kept in mind for use in the near future [52]. The data presented in this study of 2667 patients could be useful for the enrichment of these machine learning techniques. As an example, big data has been used to redefine sepsis and clinical phenotypes in Sepsis 3 [53].

This study has limitations, mainly due to the fact that it was carried out in a single center, it was retrospective in nature, and the lack of results for patients with sepsis before the implementation of the PSC model. However, we suggest that more than 2000 patients is a significant number for assessing the effectiveness of a performance program to improve sepsis care. In addition, we believe that, in future studies, it is necessary to determine the comorbidities of patients because these will likely influence prognosis and outcome. It is also necessary to continue to develop and evaluate the usefulness of biomarkers in this field [43,54].

Like other sepsis working groups, we consider it an obligation to publish our data, as it has been shown that those who carry out these studies develop the best sepsis care programs [30,55].

## 5. Conclusions

The implementation of PSC as an organizational model for the diagnosis and treatment of patients with sepsis and septic shock decreases mortality. The variables independently associated with mortality in our study population are age, increased RR, deterioration in the level of consciousness, elevation of lactate and creatinine levels, and the origin of sepsis. We recommend the implementation of specific care models, based on organizational improvements and educational programs for patients with sepsis, adapted to the idiosyncrasies of each center. 

## Figures and Tables

**Table 1:** Evolution of the characteristics of patients included in the PSC.

	2015	2016	2017	2018	2019	2020	2021	2022	
Age in years (mean, SD)	73 ± 15	71 ± 15	72 ± 15	73 ± 15	75 ± 14	76 ± 14	76 ± 14	75 ± 15	*p* < 0.05
Gender: male (%)	59.9	56.5	59.6	58.2	61.5	60.6	59.4	61.0	n.s.
Activation in the emergency department (%)	79.7	77.7	85.7	76.5	70.4	80.1	85.6	70.8	*p* < 0.05
RH lpm (average, SD)	103 ± 25	98 ± 25	104 ± 23	102 ± 24	101 ± 23	98 ± 22	101 ± 59	97 ± 23	*p* < 0.05
RR rpm (average, SD)	-	-	27 ± 8.7	27 ± 5.9	27 ± 12	28 ± 6.3	27 ± 6.9	26 ± 7.8	*p* < 0.05
SAP mmHg (mean, SD)	108 ± 28	106 ± 27	102 ± 25	99 ± 25	98 ± 24	101 ± 27	101 ± 27	101 ± 26	*p* < 0.05
SpO_2_ (average, SD)	-	-	93 ± 5.6	93 ± 5.7	93 ± 5.4	93 ± 5.3	93 ± 4.7	93 ± 5.2	n.s.
Temperature °C (average, SD)	-	-	37.4 ± 1.2	37.4 ± 1.2	38.4 ± 19	37.4 ± 2.9	37.3 ± 1.3	37.2 ± 1.2	n.s.
Low level of consciousness (%)	-	-	35.2	32.6	42.1	46.7	41.0	24.6	*p* < 0.05
Lactate mmol/L (mean, SD)	3.4 ± 3.1	2.8 ± 2.1	3.3 ± 2.6	3.2 ± 2.2	3.1 ± 2.2	3.3 ± 2.3	3.2 ± 2.5	3.1 ± 2.52	n.s.
Procalcit. mg/dL (mean, SD)	15 ± 27	9 ± 19	15 ± 26	11 ± 20	11 ± 22	13 ± 24	14 ± 26	14 ± 25	n.s.
Creatinine mg/dL (mean, SD)	1.7 ± 1.1	1.7 ± 1.8	1.2 ± 1.1	1.3 ± 1.2	1.3 ± 1.0	1.1 ± 0.6	1.8 ± 1.2	1.7 ± 1.3	*p* < 0.05
Amines, first 6 h (%)	33.5	28.6	29.2	25.3	28.3	26.1	23.7	31.7	n.s.
Antibiotics, first 6 h (%)	76.3	99.5	100	99.7	99.7	100	100	99.2	*p* < 0.05
Blood cultures collected (%)	97.8	94.4	90.8	93.9	88.6	91.6	94.3	89.4	*p* < 0.05
Positive blood cultures (%)	13.4	11.9	36.9	34.7	36.9	38.5	43.2	37.8	*p* < 0.05
ICU admission (%)	32.3	37.5	30.0	33.0	30.3	29.2	23.1	38.0	*p* < 0.05
SOFA (median, SD)	7.3 ± 3.6	5.2 ± 2.9	3.6 ± 1.8	3.9 ± 2.2	4.1 ± 2.2	4.4 ± 2.4	4.6 ± 2.7	1.4 ± 1.2	*p* < 0.05

SD: standard deviation; HR: heart rate; RR: respiratory rate; SAP: systolic arterial pressure; SpO_2_: peripheral oxygen saturation; procalcit: procalcitonin; ICU: intensive care unit; SOFA: sequential organ failure assessment; n.s.: not significant.

**Table 2:** Bivariate and multivariate analysis of factors associated with 30-day mortality.

	Bivariate Analysis		Multivariate Analysis	
	OR (CI 95%)	Significance	OR (CI 95%)	Significance
**Age > 70**	2.32 (1.86–2.89)	***p*** ** < 0.05**	2.40 (1.45–4.00)	***p*** ** < 0.05**
**Sex (% male)**	1.21 (1.02–1.42)	***p*** ** < 0.05**	0.73 (0.51–1.06)	n.s.
**RH (mean bpm)**	102 ± 26 vs. 100 ± 32	n.s.		
**RR > 22 rpm**	1.72 (1.18–2.51)	***p*** ** < 0.05**	2.03 (1.14–3.61)	***p*** ** < 0.05**
**SAP < 100 mmHg**	1.26 (1.05–1.51)	***p*** ** < 0.05**	1.52 (1.00–2.31)	n.s.
**SpO_2_ < 90%**	1.52 (1.25–1.87)	***p*** ** < 0.05**	1.23 (0.84–1.81)	n.s.
**Low level of consciousness**	2.66 (2.16–3.28)	***p*** ** < 0.05**	2.83 (1.95–4.12)	***p*** ** < 0.05**
**Serum lactate > 2 mmol/L**	2.16 (1.74–2.67)	***p*** ** < 0.05**	2.46 (1.58–3.81)	***p*** ** < 0.05**
**Procalcitonin > 2 mg/dL**	1.09 (0.91–1.30)	n.s.		
**Creatinine > 1.6**	1.65 (1.39–1.95)	***p*** ** < 0.05**	1.55 (1.06–2.26)	***p*** ** < 0.05**
**Amines, first 6 h**	1.65 (1.40–1.94)	***p*** ** < 0.05**	0.67 (0.43–1.02)	n.s.
**Antibiotic therapy, first 6 h**	0.60 (0.43–0.82)	***p*** ** < 0.05**	0.91 (0.12–6.89)	n.s.
**Blood cultures collected**	0.74 (0.56–0.97)	***p*** ** < 0.05**	0.69 (0.36–1.35)	n.s.
**Positive blood cultures**	0.95 (0.80–1.13)	n.s.		
**Origin of sepsis**		***p*** ** < 0.05**	0.87 (0.78–0.99)	***p*** ** < 0.05**
**Activation in the emergency department**	0.76 (0.64–0.91)	***p*** ** < 0.05**	0.82 (0.47–1.43)	n.s.
**Admission to ICU**	1.08 (0.91–1.29)	n.s.		
**Days in ICU (average)**	2.8 ± 6.6 vs. 3.3 ± 11	n.s.		

OR: odds ratio; IQ: confidence interval; HR: heart rate; RR: respiratory rate; SAP: systolic arterial pressure; SpO_2_: peripheral oxygen saturation; ICU: intensive care unit; n.s.: not significant.

**Table 3:** Sepsis of abdominal origin. Analysis of factors associated with 30-day mortality.

Abdominal Sepsis	Bivariate Analysis		Multivariate Analysis	
	OR (CI 95%)	Significance	OR (CI 95%)	Significance
**Age > 70**	2.58 (1.75–3.81)	***p*** ** < 0.05**	2.43 (1.46–4.05)	***p*** ** < 0.05**
**Sex (% male)**	1.05 (0.79–1.39)	n.s.		
**RH (mean bpm)**	102 ± 26 vs. 100 ± 32	n.s.		
**RR > 22 rpm**	1.33 (0.71–2.49)	n.s.		
**SAP < 100 mmHg**	1.39 (1.01–1.93)	***p*** ** < 0.05**	1.69 (0.81–3.54)	n.s.
**SpO_2_ < 90%**	1.40 (0.92–2.15)	n.s.		
**Low level of consciousness**	2.46 (1.66–3.64)	***p*** ** < 0.05**	2.61 (1.47–4.64)	***p*** ** < 0.05**
**Serum lactate > 2 mmol/L**	2.39 (1.58–3.63)	***p*** ** < 0.05**	3.77 (1.60–8.86)	***p*** ** < 0.05**
**Procalcitonin > 2 mg/dL**	1.17 (0.85–1.62)	n.s.		
**Creatinine > 1.6**	1.90 (1.43–2.53)	***p*** ** < 0.05**	2.08 (1.15–3.76)	***p*** ** < 0.05**
**Amines, first 6 h**	2.22 (1.67–2.96)	***p*** ** < 0.05**	0.14 (0.06–0.34)	***p*** ** < 0.05**
**Antibiotic therapy, first 6 h**	0.39 (0.26–0.58)	***p*** ** < 0.05**	3.70 (0.19–71.9)	n.s.
**Blood cultures collected**	0.79 (0.53–1.17)	n.s.		
**Positive blood cultures**	1.05 (0.78–1.41)	n.s.		
**Activation in the emergency department**	0.71 (0.54–0.94)	***p*** ** < 0.05**	0.55 (0.28–1.05)	n.s.
**Admission to ICU**	1.33 (1.00–1.77)	***p*** ** < 0.05**	0.35 (0.14–0.86)	***p*** ** < 0.05**

OR: odds ratio; IQ: confidence interval; HR: heart rate; RR: respiratory rate; SAP: systolic arterial pressure; SpO_2_: peripheral oxygen saturation; ICU: intensive care unit; n.s.: not significant.

**Table 4:** Sepsis of urinary origin. Analysis of factors associated with 30-day mortality.

Urinary Sepsis	Bivariate Analysis		Multivariate Analysis	
	OR (CI 95%)	Significance	OR (CI 95%)	Significance
**Age > 70**	2.52 (1.37–4.65)	***p*** ** < 0.05**	2.52 (0.53–11.9)	n.s.
**Sex (% male)**	1.04 (0.69–1.57)	n.s.		
**RH (mean bpm)**	102 ± 24 vs. 100 ± 46	n.s.		
**RR > 22 rpm**	2.99 (1.78–5.03)	***p*** ** < 0.05**	3.20 (0.86–11.9)	n.s.
**SAP < 100 mmHg**	1.10 (0.71–1.69)	n.s.		
**SpO2 < 90%**	1.84 (1.13–2.99)	***p*** ** < 0.05**	1.27 (0.55–2.95)	n.s.
**Low level of consciousness**	4.30 (2.49–7.40)	***p*** ** < 0.05**	4.32 (1.78–10.5)	***p*** ** < 0.05**
**Serum lactate > 2 mmol/L**	2.47 (1.40–4.37)	***p*** ** < 0.05**	4.03 (1.32–12.3)	***p*** ** < 0.05**
**Procalcitonin > 2 mg/dL**	1.35 (0.87–2.09)	n.s.		
**Creatinine > 1.6**	1.61 (1.07–2.43)	***p*** ** < 0.05**	1.58 (0.74–3.35)	n.s.
**Amines, first 6 h**	1.01 (0.61–1.69)	n.s.		
**Antibiotic therapy, first 6 h**	0.86 (0.29–2.53)	n.s.		
**Blood cultures collected**	0.41 (0.20–0.83)	***p*** ** < 0.05**	0.34 (0.06–1.80)	n.s.
**Positive blood cultures**	0.97 (0.65–1.45)	n.s.		
**Activation in the emergency department**	0.82 (0.46–1.49)	n.s.		
**Admission to ICU**	0.61 (0.33–1.11)	n.s.		

OR: odds ratio; IQ: confidence interval; HR: heart rate; RR: respiratory rate; SAP: systolic arterial pressure; SpO_2_: peripheral oxygen saturation; ICU: intensive care unit; n.s.: not significant.

**Table 5:** Sepsis of respiratory origin. Analysis of factors associated with 30-day mortality.

Respiratory Sepsis	Bivariate Analysis		Multivariate Analysis	
	OR (CI 95%)	Significance	OR (CI 95%)	Significance
**Age > 70**	2.57 (1.71–3.85)	***p*** ** < 0.05**	3.15 (1.62–6.11)	***p*** ** < 0.05**
**Sex (% male)**	1.16 (0.87–1.55)	n.s.		
**RH (mean bpm)**	103 ± 25 vs. 103 ± 24	n.s.		
**RR > 22 rpm**	1.14 (0.58–2.26)	n.s.		
**SAP < 100 mmHg**	1.27 (0.93–1.73)	n.s.		
**SpO_2_ < 90%**	1.51 (1.09–2.11)	***p*** ** < 0.05**	1.48 (0.89–2.47)	n.s.
**Low level of consciousness**	2.49 (1.78–3.50)	***p*** ** < 0.05**	2.66 (1.61–4.42)	***p*** ** < 0.05**
**Serum lactate > 2 mmol/L**	1.68 (1.22–2.32)	***p*** ** < 0.05**	1.67 (0.99–2.88)	n.s.
**Procalcitonin > 2 mg/dL**	1.00 (0.71–1.40)	n.s.		
**Creatinine > 1.6**	1.58 (1.17–2.12)	***p*** ** < 0.05**	1.23 (0.71–2.21)	n.s.
**Amines, first 6 h**	1.40 (1.03–1.91)	***p*** ** < 0.05**	0.48 (0.26–0.91)	***p*** ** < 0.05**
**Antibiotic therapy, first 6 h**	0.81 (0.38–1.73)	n.s.		
**Blood cultures collected**	0.84 (0.49–1.43)	n.s.		
**Positive blood cultures**	1.09 (0.79–1.50)	n.s.		
**Activation in the emergency department**	0.84 (0.58–1.22)	n.s.		
**Admission to ICU**	0.90 (0.63–1.28)	n.s.		

OR: odds ratio; IQ: confidence interval; HR: heart rate; RR: respiratory rate; SAP: systolic arterial pressure; SpO_2_: peripheral oxygen saturation; ICU: intensive care unit; n.s.: not significant.

## Data Availability

The raw data supporting the conclusions of this article will be made available by the corresponding author (rmendezh@salud.madrid.org) on request.
